# Association Between *Gardnerella vaginalis* Vaginolysin Level and Clinical Symptoms of Bacterial Vaginosis

**DOI:** 10.3390/microorganisms14020347

**Published:** 2026-02-02

**Authors:** Jiuming Li, Xiaoqi Zhu, Danhong Peng, Xuening Zhang, Lei Ba, Bei Wang, Xiang Hong

**Affiliations:** 1 Key Laboratory of Environmental Medicine and Engineering of Ministry of Education, Department of Epidemiology and Health Statistics, School of Public Health, Southeast University, Nanjing 210009, China; 2 Department of Obstetrics and Gynecology, Zhongda Hospital, School of Medicine, Southeast University, Nanjing 210009, China; 3 National Health Commission Key Laboratory of Contraceptives Vigilance and Fertility Surveillance, Nanjing 210036, China; jasonzxn@126.com (X.Z.);; 4 Jiangsu Provincial Medical Key Laboratory of Fertility Protection and Health Technology Assessment, Nanjing 210036, China; 5 Jiangsu Health Development Research Center, Nanjing 210036, China

**Keywords:** bacterial vaginosis, *Gardnerella vaginalis*, vaginolysin, vaginal microecology, clinical symptom scores, dose–response relationship

## Abstract

This study examined the role of vaginolysin (VLY), a virulence factor of the bacterium *Gardnerella vaginalis* (GV), in bacterial vaginosis (BV). In a group of 112 women with BV (diagnosis on the Nugent scale ≥7 points) and 122 control cases with normal microbiota, VLY levels, the state of the vaginal microecology (colposcopy, laboratory markers, pH), GV genotypes (clades 1–4), and clinical symptoms were assessed. It was found that GV also occurs in healthy women, but VLY levels are significantly higher in BV and correlate with inflammatory markers (e.g., leukocyte esterase) and symptom severity. However, the relationship is nonlinear: low and moderate VLY levels have little effect on symptoms, while high levels cause a sharp increase in symptoms. Thus, VLY is potentially important for the pathophysiology and clinical assessment of BV.

## 1. Introduction

Bacterial vaginosis (BV) is a vaginal dysbiosis affecting women of reproductive age, characterized by loss of vaginal lactobacilli dominance, elevated pH, and overgrowth of multiple anaerobic bacteria [1,2]. The clinical presentation of BV is highly heterogeneous: some infected individuals may have no obvious subjective symptoms, while others experience discomfort such as abnormal discharge, odor (typically a “fishy smell”), vulvar itching, or burning [3,4]. The presence or absence of symptoms is closely related to the route of detection, healthcare-seeking behavior, treatment decisions, and patient quality of life. However, systematic evidence regarding the epidemiological characteristics of BV symptoms and the relationship between symptom type/severity and vaginal microbiological features (including dominant flora and inflammatory markers) remains limited [5,6,7]. There is an urgent need to integrate clinical phenotypes with microbiological and molecular virulence factor levels to guide diagnosis, treatment, and risk assessment.

BV diagnosis commonly relies on the Nugent score and Amsel criteria, which emphasize microbiological alterations and clinical manifestations, respectively. However, both methods exhibit limitations in sensitivity, specificity, and consistency with inflammation/clinical severity. Consequently, there remains a lack of biomarkers that objectively reflect vaginal microbiological imbalance while quantifying disease activity and predicting clinical outcomes.

Research indicates that bacterial biofilms frequently form on the vaginal wall in BV, with *Gardnerella vaginalis* (GV) often colonizing as the dominant species. GV exhibits high detection rates in BV cases; during BV, it proliferates significantly (with markedly elevated colony counts) and expresses multiple potential virulence factors, such as enhanced adhesion and biofilm formation capabilities, along with secretion of the cholesterol-dependent cytotoxin vaginolysin (VLY) [2,8,9]. Although GV is widely accepted as a common pathogen in BV, it can also colonize asymptomatic women. Consequently, the precise timing and mechanism of its pathogenicity remain incompletely understood, highlighting the need for further investigation into its key virulence factors.

VLY is a cholesterol-dependent cytotoxin (CDC) secreted by GV, with its receptor being the complement-regulating protein CD59 on human cell surfaces [2,8]. In vitro studies have demonstrated that VLY lyses human erythrocytes and epithelial cells, activates the intracellular p38 MAPK pathway, and induces the secretion of proinflammatory factors such as IL-8. Furthermore, VLY’s membrane-permeating activity may be enhanced in the vaginal alkaline environment, and elevated VLY-related signals are frequently detected in clinical samples from BV patients [2,8]. Nevertheless, several critical questions regarding VLY remain unresolved: systematic evidence linking quantitative levels of VLY in vaginal secretions to patient symptoms and local inflammatory markers is lacking; how sequence and expression variations among different GV subpopulations or genetic species influence toxicity and clinical manifestations remain under-evaluated. For instance, sequence comparisons indicate that among five VLY types, Type 1 correlates with increased symptoms while Type 2 correlates with elevated GV abundance, though clinical implications require further validation [7,10]. Concurrently, there is a lack of clinically validated quantitative VLY detection methods suitable for large-scale sample screening. While recent advances in GV genetic manipulation techniques have provided new tools for functional studies, a significant translational gap persists between basic discoveries and clinical application [11].

Against this backdrop, this case–control study aims to systematically integrate vaginal microbiological indicators, GV clade/genus species profiles, and VLY expression/abundance data. It specifically evaluates correlations between VLY and clinical symptoms (presence, type, and severity). By integrating clinical phenotypes with microbiological and molecular virulence factor levels, we aim to address gaps in assessing BV clinical severity and preliminarily evaluate the feasibility and limitations of VLY as a biomarker for BV diagnosis or disease activity assessment.

## 2. Method

### 2.1. Study Population

This study is a case–control study. The study population consists of women who visited the gynecology outpatient department of Zhongda Hospital, Southeast University between October 2024 and May 2025. Inclusion criteria for the case group (BV group) are as follows: diagnosis of bacterial vaginosis based on Nugent score (≥7 points), women aged 18–80 years, and informed consent. Exclusion criteria included concomitant vaginal infections (candidal vaginitis, HPV), recent (within 4 weeks) antibiotic or hormone therapy, and immunocompromised status. Control group inclusion criteria: normal vaginal microbiota, Nugent score < 4, with other criteria identical to the case group. Exclusion criteria: same as the case group. Ultimately, 112 cases were included in the BV group and 122 in the control group. All participants signed informed consent forms. This study was reviewed and approved by the Ethics Committee of Zhongda Hospital (Ethics Approval No.: 2023ZDSYLL349). General demographic data (age, height, weight, BMI, marital status, occupation, past medical history, etc.), menstrual cycle status, and history of previous pregnancies and miscarriages were collected for both groups.

### 2.2. Clinical Symptom Assessment

Participants’ bacterial vaginosis-related clinical symptoms were comprehensively evaluated using a standardized symptom scoring system. The presence and severity of four primary symptoms—abnormal vaginal discharge, fishy odor, itching, and burning sensation—were comprehensively considered to assign an overall severity rating: 0 points (asymptomatic), 1 point (mild), 2 points (moderate), 3 points (severe). This total score was used in the study protocol to quantify the overall severity of BV symptoms.

### 2.3. Vaginal Microecology Assessment

Vaginal microecology status was evaluated using a combination of colposcopy and laboratory testing:

Vaginal Cleanliness Grading: Vaginal smear preparations were graded according to the cleanliness classification standards (I–IV) recommended in the National Clinical Laboratory Procedures. Additional details are provided in the Supplementary Materials (Supplementary Data S1).

Lactic Acid Bacteria Count: Gram- positive rods were counted via Gram staining.

Clue Cells: microscopic examination was carried out to determine the presence of clue cells.

Biochemical indicators: hydrogen peroxide (H_2_O_2_) production and leukocyte esterase activity in vaginal secretions were detected using test strips or microscopy, and vaginal pH was measured.

### 2.4. GV Genotyping

GV subpopulation genotyping employs a molecular detection method based on multiplex PCR. Following the genotyping protocol reported by Balashov, S.V et al. [12], specific primers were designed to amplify distinct GV gene fragments. Based on amplification band patterns, GV was classified into four major clades (clade 1–4). PCR reaction systems and amplification conditions were strictly executed according to standardized protocols to ensure genotyping accuracy (Supplementary Data S2).

### 2.5. VLY Detection

#### 2.5.1. Preparation of VLY Recombinant Protein and Polyclonal Antibody

Primers were designed based on the VLY protein amino acid sequence 321–491, followed by gene synthesis. The gene was cloned into an expression vector and expressed in *E. coli*. High-purity VLY recombinant protein (approximately 36.8 kDa) was obtained via Ni^2+^ affinity chromatography and used as the immunogen. Healthy female New Zealand White rabbits were immunized multiple times (initially with Freund’s complete adjuvant, followed by Freund’s incomplete adjuvant) to generate VLY polyclonal antibodies. Serum titer was determined via indirect ELISA, showing ≥1:512 K titer, confirming successful antibody production. Further purification of the antibody was achieved via protein A affinity chromatography, and its specificity was validated by Western blot analysis. Results confirmed the antibody’s specific recognition of VLY protein (Supplementary Data S3).

#### 2.5.2. Quantitative Detection of VLY

Vaginal secretion samples were centrifuged to remove cells and impurities, followed by quantitative detection of VLY protein concentration using a sandwich ELISA method based on anti-VLY polyclonal antibodies. Experimental conditions followed the latest literature [13]: Anti-VLY antibodies were coated onto a 96-well high-binding microplate (4 °C, overnight). After blocking, processed vaginal secretion samples were added. HRP-labeled secondary antibodies were added, color was developed with TMB, the reaction was stopped, and then OD was measured at 450 nm. VLY concentration (ng/mL) in samples was calculated using a standard curve (serial dilutions of recombinant VLY protein). Log-transformed values were used for subsequent statistical analysis.

## 3. Statistical Analysis

Statistical analysis was performed using R software version 4.3.0. Continuous variables, after normality testing, were expressed as mean ± standard deviation or median (interquartile range). Comparisons between two groups were performed using independent samples *t*-tests or Mann–Whitney U tests. Categorical variables were expressed as frequencies or percentages, and comparisons between groups were performed using chi-squared tests or Fisher’s exact tests. Spearman’s rank correlation analysis was employed to evaluate the relationship between VLY levels and vaginal microecology indicators as well as symptom scores. Symptom scores served as the dependent variable, while VLY levels and potential influencing factors (e.g., age, BMI, colposcopy findings) acted as independent variables. After screening via univariate linear regression, variables were incorporated into a multivariate linear regression model. The significance level was set at α = 0.05.

## 4. Results

Subject Characteristics. There were no significant differences between the two groups in demographic indicators such as age, height, weight, and BMI (*p* > 0.05). The BV group comprised 112 BV patients, while the control group included 122 healthy controls. Symptom scores were significantly higher in the BV group than in the control group (*p* < 0.001). The BV group exhibited markedly higher proportions of mild, moderate, and severe symptoms compared to the control group, where most participants were asymptomatic. Diabetes mellitus was present in seven cases (5.7%) of the control group but none in the BV group (*p* = 0.029). No significant differences were observed between groups regarding other preexisting conditions (e.g., hypertension), marital status, or occupational distribution (*p* > 0.05) (Table 1).

Vaginal Microecological Indicators. In the BV group, 98.2% of cases exhibited Grade IV vaginal cleanliness, whereas the control group predominantly showed normal cleanliness (*p* < 0.001). Vaginal Gram staining revealed a marked reduction in Gram- positive rods, with no detection of Gram- positive rods (Lactobacillus) in 91.1% of BV cases, indicating Lactobacillus depletion. All BV group samples tested positive for clue cells (100%). The mean vaginal pH was 4.8 ± 0.1, significantly higher than the normal acidic range. These alterations align with the classic microbiological dysbiosis pattern of BV (Table 2).

**Figure 1:**
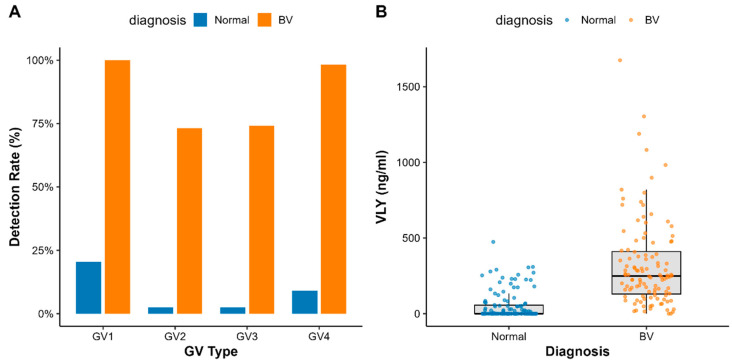
GV clade distribution and VLY expression (**A**) Detection and distribution of GV clades (clades 1–4) in the BV group and the control group; (**B**) Comparison of VLY concentrations in vaginal secretions between BV patients and healthy controls.

The distribution results of GV subtypes indicate that GV vaginalis can be detected even in healthy control groups, suggesting it may exist as a commensal bacterium in the vaginas of some healthy women. As shown in Figure 1A, multiple GV subtypes were detected in both BV and non-BV groups. Although VLY secretion was detected in both groups, the expression level of VLY was significantly higher in the BV group than in the control group (Figure 1B). This result supports the notion that VLY is closely associated with BV.

Correlation between the number of GV subtypes and symptoms. Using Spearman’s correlation analysis, no significant correlation was observed between multi-subtype GV infection (the number of GV subtypes detected) and symptom scores (correlation coefficient r ≈ 0.037, *p* = 0.70), indicating that the number of GV subtypes itself is not a major factor affecting the severity of BV symptoms (Table 3).

**Figure 2:**
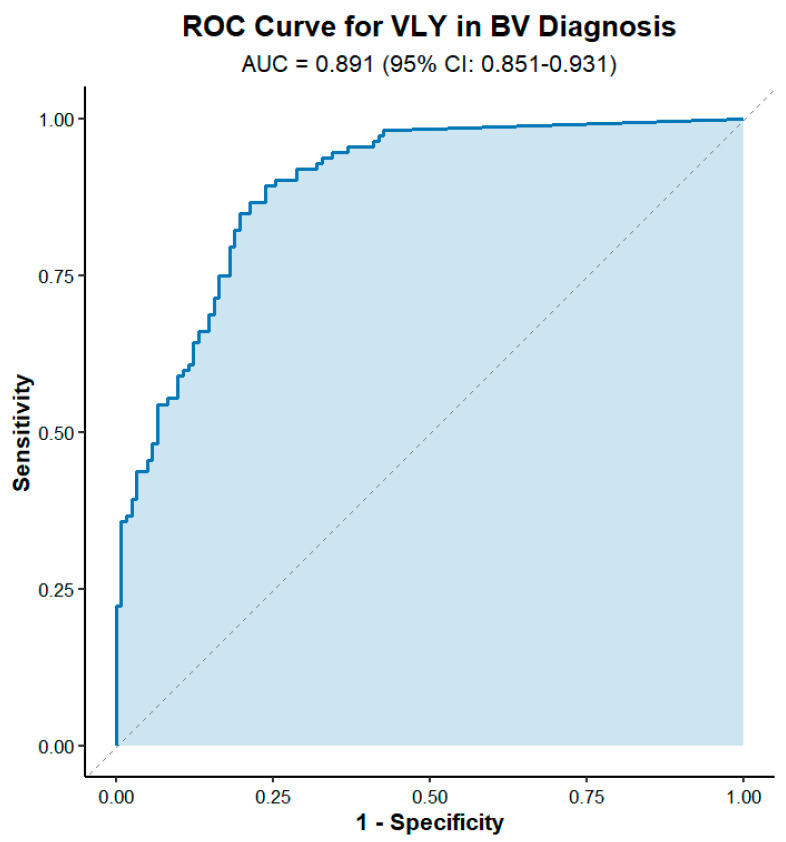
Diagnostic value of VLY for BV.

To evaluate the potential of VLY as a diagnostic biomarker, we performed ROC curve analysis on VLY levels. Results showed that VLY demonstrated an AUC of 0.889 (95% CI: 0.851–0.931) in distinguishing BV from non-BV, indicating high diagnostic accuracy (Figure 2). Further analysis identified the optimal cutoff value as 63.7 (95% CI: 28.8–93.4) ng/mL, where sensitivity and specificity were balanced at 0.893 and 0.762, respectively. Therefore, this threshold was subsequently adopted as the criterion for distinguishing VLY-positive from VLY-negative cases in this study.

**Figure 3:**
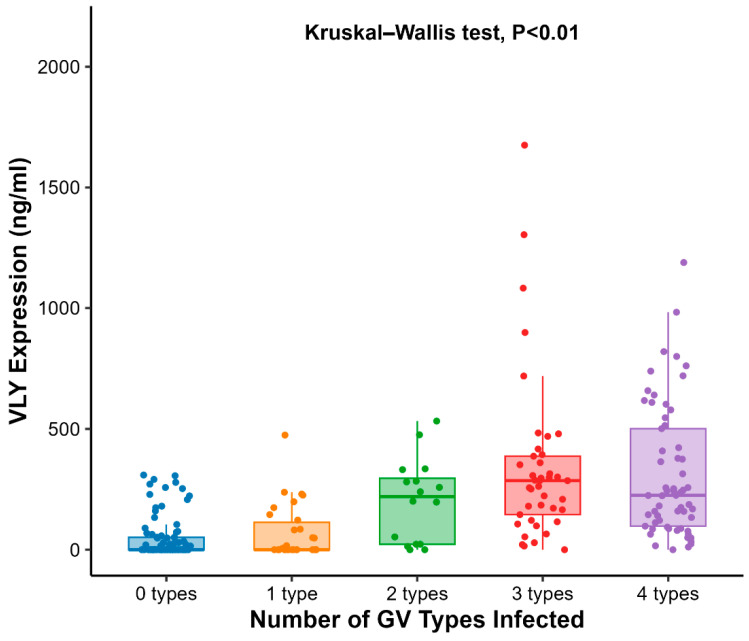
VLY levels by GV clade number.

We further analyzed VLY expression levels in patients infected with different numbers of GV subtypes. Results showed a significant upward trend in VLY expression with increasing numbers of GV subtypes. The median VLY expression in the multi-subtype co-infection group was markedly higher than in the single-subtype group, suggesting that different GV subtypes may synergistically enhance VLY production. The boxplot in Figure 3 displays the distribution of VLY expression across groups with 0–4 subtypes, with the median and quartiles represented by the box. Comparisons between groups were performed using the Kruskal–Wallis test, revealing a statistically significant difference between the multi-subtype group and the single-subtype group (*p* < 0.01) (Figure 3).

Within the BV group, analysis revealed a significant correlation between leukocyte esterase (LE) levels and VLY levels among vaginal microecology indicators: patients with LE-negative results exhibited the lowest median VLY levels (222.8 ng/mL), significantly lower than the weakly positive and moderately positive LE groups (*p* = 0.027). Furthermore, the VLY positivity rate calculated using the threshold of 63.7 ng/mL determined by ROC curve analysis was only 82.6%, markedly lower than that of other groups (97%) (*p* = 0.017), suggesting that VLY expression may be closely associated with local inflammatory status. No significant differences were observed between VLY levels and other microbiological indicators (cleansing grade, H_2_O_2_ production) (*p* > 0.05). Regarding clinical symptom scores, VLY levels increased with symptom severity: the median was 227.5 ng/mL in the asymptomatic group, while the median reached 609.5 ng/mL in the severe symptom group (n = 3), with a statistically significant intergroup difference (*p* = 0.008). This suggests VLY may reflect the clinical severity of BV . VLY expression was significantly lower in the LE-negative group compared to the LE-positive group, and LE-negative patients exhibited markedly lower VLY positivity rates. Higher symptom scores correlated with elevated VLY levels (all *p* values < 0.05) (Table 4).

VLY showed linear correlations with symptom scores and pH. After taking the logarithm of VLY for linear regression analysis, results indicated that VLY levels were positively correlated with clinical symptom scores (β = 0.27, *p* = 0.04) and negatively correlated with vaginal pH (β = −0.047, *p* = 0.028), that is, increased VLY concentrations were associated with more severe symptoms and a tendency toward lower pH (Table 5).

Univariate linear regression analysis revealed a significant positive correlation between VLY levels and bacterial vaginosis (BV) symptom severity scores (β = 0.118, 95% CI: 0.00562–0.23, *p* = 0.0397). Age showed a significant negative correlation with symptom scores (β = −0.0116, 95% CI: −0.0231 to −0.00011, *p* = 0.0479), while hydrogen peroxide levels exhibited a significant negative correlation (β = −1.48, 95% CI: −2.50 to −0.457, *p* = 0.00497). Other variables including BMI, vaginal pH, cleanliness, leukocyte esterase, and bacterial status did not reach significance in univariate analysis (all *p* > 0.05).

In the multivariate linear regression model, after adjusting for age, BMI, pH, cleanliness, bacterial status, hydrogen peroxide, and leukocyte esterase, age remained significantly negatively correlated (β = −0.0127, 95% CI: −0.0252 to −0.00027, *p* = 0.0453), while hydrogen peroxide levels also maintained a significant negative correlation (β = −2.13, 95% CI: −4.26 to −0.00546, *p* = 0.0494). In contrast, while the linear effect of VLY remained positive, its statistical significance weakened (β = 0.083, 95% CI: −0.0287 to 0.195, *p* = 0.144), suggesting its relationship with symptom scores may not be linear or may be partially influenced by other microecology indicators (Table 6).

**Figure 4:**
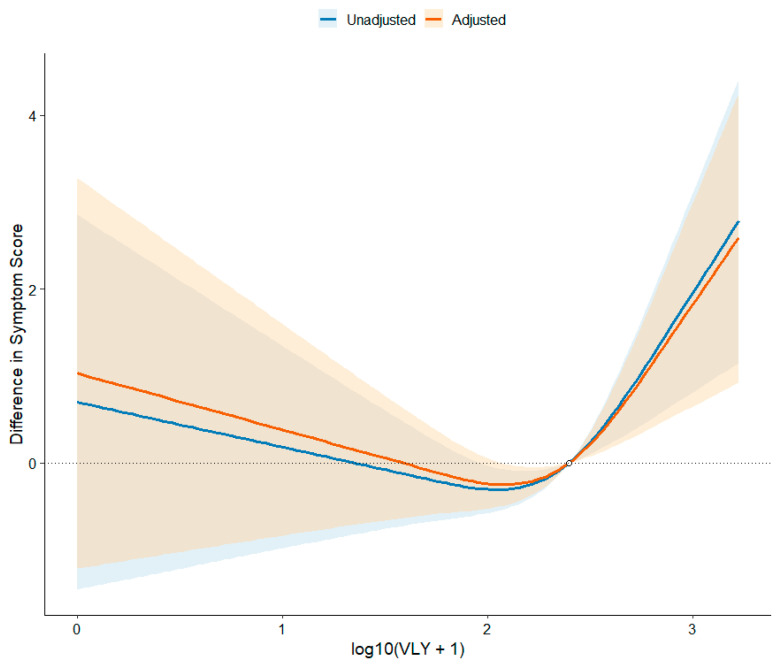
Dose–response relationship between VLY and symptoms.

To further elucidate the dose–response relationship between VLY concentration and bacterial vaginosis symptom severity, we employed a three-knot restricted cubic spline (RCS) model for analysis [14]. In the unadjusted model, log-transformed VLY concentration showed a significant overall association with symptom severity (overall *p* = 0.0032), and the nonlinear term was also statistically significant (*p* = 0.0033), indicating a clear nonlinear relationship between the two. After adjusting for age, BMI, vaginal pH, cleanliness grading, bacterial status, hydrogen peroxide levels, and leukocyte esterase activity, the correlation between VLY and symptom scores remained statistically significant (overall spline *p* = 0.0096). The nonlinear component of the adjusted spline curve remained statistically significant (*p* = 0.0049), indicating that the nonlinear dose–response pattern persisted independently of potential confounders. Using the median logVLY value as the reference point, the adjusted RCS curve demonstrated a monotonically increasing trend in predicted symptom scores with rising VLY concentrations. The curve slope steepens at higher VLY levels, suggesting disproportionately worsening symptom severity in patients with elevated VLY. This nonlinear pattern indicates that low-level increases in VLY correlate with relatively mild symptom changes, whereas higher VLY levels correspond to a steep increase in symptom burden. Collectively, these findings suggest that VLY may be associated with BV symptomatology in a nonlinear manner, further reinforcing its potential value as a biomarker for quantifying disease activity (Figure 4).

In summary, this study found that VLY concentration in the vaginal secretions of BV patients was significantly correlated with clinical symptoms and local inflammatory markers, whereas the number of GV subtypes infected showed no clear association with symptoms. VLY expression appears to be influenced by multiple GV subtype infections, yet its independent association with symptoms suggests VLY may serve as a potential biomarker for assessing BV disease severity.

## 5. Discussion

This study systematically evaluated the relationship between VLY levels in vaginal secretions and clinical characteristics of bacterial vaginosis (BV) based on case–control data from a Chinese population. Results showed that VLY concentrations were significantly higher in BV patients than in healthy controls and were positively correlated with local inflammatory markers (e.g., leukocyte esterase activity) and clinical symptom scores. This finding aligns with VLY’s known biological mechanism as a cholesterol-dependent cytotoxin (CDC) [15]. Although BV is classically associated with elevated vaginal pH, the relationship between pH and specific virulence factors such as VLY may be influenced by sampling variability, limited subgroup size, and complex host–microbe interactions. Therefore, the correlations observed in regression analyses should be interpreted cautiously and considered hypothesis-generating rather than definitive. Previous studies indicate that VLY lyses vaginal epithelial cells, activates the p38 MAPK signaling pathway, and induces the production of proinflammatory factors such as IL-8 and IL-1β, thereby amplifying the local inflammatory response [1,8,16]. Pleckaityte et al. demonstrated that over 50% of BV patients produce IgA antibodies against VLY alongside elevated IL-1β, further supporting VLY’s involvement in BV inflammation [1]. In this study, lower VLY levels in leukocyte esterase-negative patients may reflect reduced inflammatory intensity; conversely, elevated VLY correlated with worsening subjective symptoms (e.g., vaginal odor, increased discharge), suggesting its potential as an adjunctive indicator for assessing BV symptom severity.

Regarding GV subtypes, this study observed higher VLY expression during polyclonal infections, implying potential synergistic or antagonistic interactions between different GV clones that collectively influence overall virulence output. This finding aligns with BV’s characteristics of multispecies coexistence and biofilm formation. Previous studies have reported that BV-associated bacteria can synergistically enhance pathogenicity within biofilms [1]. For instance, *Enterococcus faecalis* and *Actinomyces neuii* can upregulate GV’s VLY expression, while Lactobacillus crispatus can suppress VLY expression and reduce its cytotoxicity [17]. However, no significant association was observed between GV subtype abundance and symptom severity in this study, consistent with Pleckaityte et al.’s findings that VLY production in clinical isolates does not directly correlate with GV genotype or biotype [4]. Symptom complexity may be jointly regulated by host immune status, microbiota composition, and microbial interactions.

Our findings partially align with those of Janulaitiene et al.: clade 1 and clade 2 subtypes are more strongly associated with BV, while clade 4 is more prevalent in healthy individuals [5]. This study observed elevated VLY levels during multi-subtype infections, suggesting enhanced population virulence of BV-associated subtypes. However, the number of GV subtypes failed to explain differences in symptom scores, potentially indicating toxicity variations due to VLY sequence diversity. Recent sequence studies suggest VLY exhibits multiple virulence types, with Type 1 associated with symptom severity and Type 2 primarily affecting GV abundance [10,17]. Furthermore, genetic modification studies demonstrate that VLY-knockout GV mutants exhibit significantly reduced lytic activity against cervical tissue in vitro, confirming VLY’s central role in BV pathogenesis [11].

Further statistical analysis in this study employed a restricted cubic spline model, revealing a significant nonlinear relationship between VLY levels and BV symptoms. Results demonstrated that the nonlinear component remained statistically significant both unadjusted and after adjusting for potential confounders (including age, BMI, pH, cleanliness grade, bacillary status, hydrogen peroxide, and leukocyte esterase), suggesting robustness of this nonlinear dose–response pattern [18]. Specifically, VLY was associated with a gradual increase in symptom scores across low-to-moderate concentration ranges, whereas symptom severity increased steeply at higher VLY levels, suggesting a potential dose-threshold effect or accelerated toxic response at high doses. This aligns strongly with experimental studies on CDC toxins: low doses primarily activate signaling pathways, while high doses directly induce membrane lysis and intense inflammatory cascades [2,3]. Concurrently, BV biofilm microbiota may exhibit a stepwise increase in virulence characteristics upon reaching certain abundance thresholds, further explaining the disproportionate worsening of symptoms. Notably, while VLY exhibited non-significant linear effects in multivariate linear regression, its effects persisted in nonlinear models. This suggests that the association between VLY and symptoms may not be fully captured by linear models and is better described by nonlinear dose–response relationships.

Collectively, this study highlights a significant association between VLY and BV pathogenesis and supports its investigation as a candidate biomarker. Significantly elevated VLY levels in BV correlate with symptom severity and exhibit a robust nonlinear dose–response relationship, suggesting its potential utility as a research tool for severity assessment. However, as noted in the limitations, its clinical application for diagnosis or monitoring requires external validation of the proposed cutoff and assessment of feasibility against current standards. Existing literature confirms a close association between in vivo VLY levels and BV biological activity [1,19], though low-level VLY detection in healthy women indicates its specificity requires further validation [6]. Furthermore, studies indicate that VLY acts differentially on distinct receptor surfaces of cervical epithelium: high concentrations strongly induce inflammation on the basal surface, while exposure on the apical surface may suppress neutrophil recruitment [1,9,20]. This may explain the clinical paradox where BV exhibits marked inflammatory factor elevation yet lacks typical neutrophil infiltration.

This study has several limitations. First, as a single-center study with a small sample size, the generalizability of the findings may be restricted. Despite the observed trends across symptom severity and microecological subgroups, several analyses involved very small sample sizes. Analyses involving very small subgroups (e.g., n ≤ 3) were exploratory in nature and are presented descriptively. The limited sample size precludes robust statistical inference, and these findings should be interpreted with caution. Second, the study did not simultaneously evaluate the interactions between VLY and other key GV virulence factors (such as sialidases and extracellular proteases), thus failing to comprehensively reflect the overall virulence spectrum of GV [4]. The VLY cutoff value was derived and evaluated within the same dataset, which may lead to overfitting. Therefore, this threshold should be considered preliminary and requires validation in independent cohorts before clinical application. Finally, the cross-sectional design precludes establishing temporal causality between VLY levels and symptom progression.

Future studies should validate VLY’s sensitivity, specificity, and clinical utility in BV classification across multicenter, large-scale populations. Combining whole-genome analysis of GV could elucidate functional differences among virulence types. Animal models or organoid systems should investigate VLY’s dose–response mechanisms and dynamic role in BV progression, providing theoretical foundations for its use as a diagnostic or therapeutic target [21].

## 6. Conclusions

In summary, this case–control study reveals that vaginal levels of vaginolysin (VLY), a key virulence factor of *Gardnerella vaginalis*, are significantly elevated in patients with bacterial vaginosis (BV). Our findings demonstrate a significant nonlinear association between VLY concentration and the severity of clinical symptoms, suggesting a potential dose-threshold relationship. Furthermore, VLY levels correlated positively with the inflammatory marker leukocyte esterase but not with the simple diversity of *G. vaginalis* clades, indicating that pathogenic output may be more critical than pathogen diversity in driving symptoms.

These results position VLY as a promising candidate biomarker linked to the clinical and inflammatory phenotype of BV. However, the cross-sectional design of this study precludes causal inference, and the proposed diagnostic threshold requires external validation. Future large-scale, longitudinal studies are necessary to confirm the observed dose–response relationship, to evaluate the clinical utility and cost-effectiveness of VLY testing alongside existing diagnostic methods, and to explore its potential in stratifying disease severity or monitoring treatment response.

## Figures and Tables

**Table 1:** Characteristics of participants.

Factors	Normal	BV	*p*-Value
	n = 122	n = 112	
Age (years, Mean ± SD)	41.3 ± 11.7	41.6 ± 11.7	0.856
Height (cm, Mean ± SD)	161.3 ± 5.9	160.9 ± 5.3	0.595
Weight (kg, Mean ± SD)	55.8 ± 9.2	57.8 ± 9.1	0.102
BMI (kg/m^2^, Mean ± SD)	21.7 ± 3.4	22.4 ± 2.9	0.094
Employment Status [n (%)]			0.296
Unemployed	76 (62.3)	78 (69.6)	
Employed	46 (37.7)	34 (30.4)	
Marriage Status [n (%)]			0.659
Unmarried	6 (4.9)	8 (7.1)	0.065
Married	116 (95.1)	104 (92.9)	
Score [n (%)]			<0.001
0	73 (59.8)	30 (26.8)	
1	36 (29.6)	62 (55.4)	
2	13 (10.7)	17 (15.2)	
3	0 (0)	3 (2.7)	
History of Diabetes [n (%)]	7 (5.7)	0 (0)	0.029
History of Hypertension [n (%)]	8 (6.6)	7 (6.2)	1
History of Miscarriage [n (%)]			0.791
0	60 (49.29)	52 (46.49)	
1	33 (27.0)	29 (25.99)	
≥2	29 (23.89)	31 (27.7)	
Menstrual cycle regularity [n (%)]			0.716
Regular	106 (86.99)	100 (89.39)	
Irregular	16 (13.1)	12 (10.7)	

**Table 2:** Vaginal microecology indicators.

Factors	BV
	n = 112
**Morphological indicators**	
**Cleanliness [n (%)]**	
Grade III	2 (1.8)
Grade IV	110 (98.2)
**Bacillus [n (%)]**	
Negative	102 (91.1)
Weakly positive	8 (7.1)
Strongly positive	2 (1.8)
**Clue cell [n (%)]**	
Negative	0 (0.0)
Positive	112 (100)
**Biochemical indicators**	
**Hydrogen peroxide [n (%)]**	
Negative	0 (0.0)
Weakly positive	8 (7.1)
Positive	104 (92.9)
**Leukocyte esterase [n (%)]**	
Weakly positive	23 (20.5)
Positive	29 (25.9)
Moderate positive	36 (32.1)
Strongly positive	24 (21.4)
**PH (Mean ± SD)**	4.8 ± 0.1

**Table 3:** Relationship between GV infections and symptoms.

GV Type Count	Score = 0 [n (%)]	Score = 1 [n (%)]	Score = 2 [n (%)]	Score = 3 [n (%)]	r	*p*
2	4 (40.0)	3 (30.0)	3 (30.0)	0 (0.0)	0.0367	0.7
3	11 (26.8)	24 (58.5)	4 (9.8)	2 (4.9)		
4	15 (24.6)	35 (57.4)	10 (16.4)	1 (1.6)		

**Table 4:** Relationship between VLY and vaginal microecological indicators and symptom scores in the BV group.

Microecological Indicators	Group (n)	Median VLY Expression (IQR)	*p* Value	VLY Positivity Rate (%) (Positive/Total)	*p* Value
Cleanliness	Grade III (n = 2)	200.2 (146.9–253.6)	0.668	100.0 (2/2)	1
	Grade IV (n = 110)	248.9 (135.1–414.7)		89.1 (98/110)	
Bacillus	Negative (n = 102)	245.4 (121.3–391.8)	0.404	88.2 (90/102)	0.517
	Weakly positive (n = 8)	407.3 (161.6–605.9)		100.0 (8/8)	
	Strongly positive (n = 2)	200.2 (146.9–253.6)		100.0 (2/2)	
Clue cells	Positive (n = 112)	248.9 (130.3–410.7)	–	89.3 (100/112)	–
Hydrogen peroxide	Weakly positive (n = 8)	407.3 (192.5–605.9)	0.147	100.0 (8/8)	0.596
	Strongly positive (n = 104)	245.4 (119.8–388.6)		88.5 (92/104)	
Leukocyte esterase	Negative (n = 23)	222.8 (100.5–388.2)	0.027	82.6 (19/23)	0.017
	Weakly positive (n = 29)	299.2 (168.6–422.2)		96.6 (28/29)	
	Moderately positive (n = 36)	269.3 (178.1–487.7)		97.2 (35/36)	
	Strongly positive (n = 24)	170.1 (60.9–268.0)		75.0 (18/24)	
Symptom score	Asymptomatic (n = 30)	227.5 (130.8–305.0)	0.008	86.7 (26/30)	0.798
	Mild (n = 62)	223.9 (107.8–410.9)		88.7 (55/62)	
	Moderate (n = 17)	300.9 (252.6–658.1)		94.1 (16/17)	
	Severe (n = 3)	609.5 (480.5–956.8)		100.0 (3/3)	

**Table 5:** Linear regression results.

Independent Variable	Dependent Variable	Beta	*p*-Value
VLY (log)	Symptom score	0.27	0.04
VLY (log)	PH	−0.047	0.028

**Table 6:** Univariate and multivariate linear regression analyses of factors associated with bacterial vaginosis symptom severity.

Variable	β (Univariate, 95% CI)	*p* (Univariate)	β (Multivariate, 95% CI)	*p* (Multivariate)
VLY (log)	0.12 (0.01, 0.23)	0.04	0.08 (−0.03, 0.20)	0.144
Age	−0.01 (−0.02, −0.00)	0.048	−0.01 (−0.03, −0.00)	0.045
BMI	−0.01 (−0.06, 0.04)	0.706	−0.02 (−0.07, 0.03)	0.436
pH	−1.05 (−2.19, 0.08)	0.068	0.44 (−0.99, 1.88)	0.542
Cleanliness	0.96 (−0.06, 1.97)	0.065	0.38 (−1.85, 2.61)	0.737
Hydrogen peroxide	−1.48 (−2.50, −0.46)	0.005	−2.13 (−4.26, −0.01)	0.049
Leukocyte esterase	−0.01 (−0.16, 0.15)	0.946	0.03 (−0.12, 0.19)	0.672
Lactobacillus	0.05 (−0.33, 0.43)	0.789	−0.30 (−1.31, 0.71)	0.559

## Data Availability

The original contributions presented in this study are included in the article/supplementary material. Further inquiries can be directed to the corresponding authors.

## Supplementary Materials

The following supporting information can be downloaded at: https://www.mdpi.com/article/10.3390/microorganisms14020347/s1, Data S1: Vaginal Cleanliness Testing and Grading Standards; Data S2: GV Genotyping Detection Primer Sequences; Data S3: Preparation, Purification, and Characterization of VLY Polyclonal Antibodies.
