# Supplementary material for: Association Between Gardnerella vaginalis Vaginolysin Level and Clinical Symptoms of Bacterial Vaginosis
Source: Microorganisms. 2026 Feb 2;14(2):347. doi: 10.3390/microorganisms14020347 (PMC12943129; doi:10.3390/microorganisms14020347)
Supplement: Supplementary file 1 [file microorganisms-14-00347-s001.zip › Supplementary Data S1.pdf]

Vaginal Cleanliness Testing and Grading Standards

1. Specimen Collection

Using a sterile swab, gently rotate to collect material from the upper third of the vaginal lateral wall, avoiding contact with the cervical os and surface mucus of the vaginal wall.

Spread the collected material evenly onto a clean microscope slide. Allow it to air dry at room temperature before performing Gram staining.

2. Microscopic Examination

Observe the stained smear under an oil immersion lens (10×100 magnification).

Key observation criteria include:

Bacilli: Primarily lactobacilli, appearing as purple Gram-positive rods.

Cocci: Gram-positive or Gram-negative cocci.

Epithelial cells: Polygonal or round cells in the background.

Leukocytes (pus cells): Morphologically intact cells with lobulated nuclei in the cytoplasm.

3. Cleanliness Grading Criteria

Vaginal cleanliness is graded as Grade I, II, III, or IV based on microscopic examination.

Grade I: Vaginal flora dominated by lactobacilli; abundant vaginal epithelial cells visible; no cocci or other contaminants; ≤5 pus cells per high-power field.

Grade II: Partial lactobacilli and vaginal epithelial cells present; >5 but ≤15 pus cells per high-power field. Grade II discharge shows significantly reduced lactobacilli and epithelial cells compared to Grade I, with some pus cells and miscellaneous bacteria visible.

Grade III: Only minimal lactobacilli and epithelial cells are present, while miscellaneous bacteria increase significantly; 15–30 pus cells per high-power field.

Grade IV: Completely opposite to Grade I, with almost no lactobacilli or vaginal epithelial cells visible. The field is dominated by miscellaneous bacteria, and pus cells exceed 30 per high-power field. 4. Precautions

Avoid sexual intercourse, baths, vaginal douching, and local medication within 24 hours prior to examination.

The smear should be of appropriate thickness, as staining quality directly affects interpretation results.
