# Supplementary material for: Association Between Gardnerella vaginalis Vaginolysin Level and Clinical Symptoms of Bacterial Vaginosis
Source: Microorganisms. 2026 Feb 2;14(2):347. doi: 10.3390/microorganisms14020347 (PMC12943129; doi:10.3390/microorganisms14020347)
Supplement: Supplementary file 1 [file microorganisms-14-00347-s001.zip › Supplementary Data S3.pdf]

Preparation, Purification, and Characterization of VLY Polyclonal Antibodies

S1.Materials

S1. 1 Test Materials and Reagents

Reagent name	Supplier	Number
YEAST EXTRACT	OXOID	LP0021
Restriction endonuclease 1	NEB	
Restriction endonuclease 2	NEB	
Pfu DNA polymerase	Life technology	12344024
T4 DNA Ligase	Roche	10481220001
DNA Recovery Kit	Tiangen	DP214
Plasmid extraction kit	Axygen	AP-MN-P-250
Competent cell	Beijing TransGen Biotech	
TRYPTONE	OXOID	LP0042
NaCl	Sinopharm Group Chemical Reagent Co., Ltd.	10019318
Kanamycin sulfate	Amresco	0408
Agar powder	Wuhan Tianyuan Huid Bio-Technology Co., Ltd.	BM0202
EDTA·2Na	Sinopharm Group Chemical Reagent Co., Ltd.	10009717
KH2PO4	Sinopharm Group Chemical Reagent Co., Ltd.	10017618
Na2HPO4	Sinopharm Group Chemical Reagent Co., Ltd.	20040618
NiSO4	Sinopharm Group Chemical Reagent Co., Ltd.	10014418

Sodium acetate	Sinopharm Group Chemical Reagent Co., Ltd.	10018718
Imidazole	Sinopharm Group Chemical Reagent Co., Ltd.	30104916
H3PO4	Sinopharm Group Chemical Reagent Co., Ltd.	10015418
ethanol	Sinopharm Group Chemical Reagent Co., Ltd.	10009218
G250	Shanghai Vocas Chemical Reagent Co., Ltd.	71011284
Syringe filter	Tianjin Jinteng Experimental Equipment Co., Ltd.	Φ25 0.22/ 0.45μm(Water)
Protein MW Marker	Thermo Scientific™	26610
rProtein A Beads	Changzhou Tiandi Renhe Biotechnology Co., Ltd.	SA012500
CNBr-activated Bestarose 4B	Biolong (Shanghai) Biotechnology Co., Ltd.	SMSAA001
Incomplete adjuvant	sigma	F5881
Complete adjuvant	sigma	F5506
Rabbit secondary antibody	Jackson	116154

S1.2 Main Instrument

Name	Producer	Model
Clean bench	AIRTECH	SW-CJ-2FD
Ultra-low temperature refrigerator	Qingdao Haier Special Electric Appliance Co., Ltd.	DW-86L626
Incubator	Shanghai Senxin Experimental Instrument Co., Ltd.	MJP-D

Shanghai Jinghong Blast Drying Oven	Shanghai Jinghong Experimental Equipment Co., Ltd.	DHG-9076A
-20 ℃Freezer	Henan Xinfei Electric Co., Ltd.	BC/BD-263HB
Benchtop high-speed refrigerated centrifuge	Cence	H2050R
Microplate reader	KHB	ST-260
Micro spectrophotometer	Merinton	SMA4000
-80 Ultra-low temperature refrigerator	Thermo	900SERIES

S2 Experimental methods

S2.1 Preparation of Prokaryotic Expression Plasmid Vectors for VLY Recombinant Protein

S2.1.1 Gene Synthesis and Vector Construction

Primers were designed based on the amino acid sequence of VLY protein 321-491 for gene synthesis. The gene sequence is as follows:

Immunogen gene sequence：

ATGAGCGATAAAATTATTCACCTGACTGACGACAGTTTTGACACGGATGTACTCAAAGCGGACGG
GGCGATCCTCGTCGATTTCTGGCAGAGTGGTGCGGTCCGTGCAAAATGATCGCCCCGATTCTGGAT
GAAATCGCTGACGAATATCAGGGCAAACTGACCGTTGCAAAACTGAACATCGATCAAAACCCTGG
CACTGCGCCGAAATATGGCATCCGTGGTATCCCGACTCTGCTGCTGTTCAAAAACGGTGAAGTGG
CGGCAACCAAAGTGGGTGCACTGTCTAAAGGTCAGTTGAAAGAGTTCCTCGACGCTAACCTGGC
CGGTTCTGGTTCTGGCCATATGCACCATCATCATCATCATTCTTCTGGTCTGGTGCCACGCGGTTCT
GGTATGAAAGAAACCGCTGCTGCTAAATTCGAACGCCAGCACATGGACAGCCCAGATCTGGGTAC
CGACGACGACGACAAGAAGGTGATCACCGGTAATATTGATACCCTGAAAGCACTGATTCAGGAAG
GCGCCAATCTGAGCACCAGTAGCCCGGCAGTTCCGATTGCATATACCACCAGCTTTGTTAAAGACA
ATGAGGTGGCCACCCTGCAGAGCAATAGTGATTATATTGAAACCAAGGTGAGCAGTTACCGCAAT
GGCTATCTGACCCTGGATCATCGCGGTGCCTATGTTGCACGCTATTATATTTATTGGGACGAATACG
GTACCGAAATTGATGGCACCCCGTATGTTCGTAGCCGCGCTTGGGAAGGCAATGGTAAATATCGTA
CCGCCCATTTTAACACCACCATTCAGTTTAAAGGCAACGTTCGTAATCTGCGTATTAAACTGGTTG
AAAAGACCGGTCTGGTGTGGGAACCGTGGCGTACAGTTTATGATCGCAGCGATCTGCCGCTGGTT

CGTCAGAGAACCATTAGTAATTGGGGTACCACCCTGTGGCCGCGTGTTGCAGAAACCGTTAAAAA

TGATCACCACCACCACCACCACTGA

Amino acid sequence：

MSDKIIHLTDDSFDTDVLKADGAILVDFWAEWCGPCKMIAPILDEIADEYQGKLTVAKLNIDQNPGTA

PKYGIRGIPTLLLFKNGEVAATKVGALSKGQLKEFLDANLAGSGSGHMHHHHHHSSGLVPRGSGMKE

TAAAKFERQHMDSPDLGTDDDDKKVITGNIDTLKALIQEGANLSTSSPAVPIAYTTSFVKDNEVATLQS

NSDYIETKVSSYRNGYLTLDHRGAYVARYYIYWDEYGTEIDGTPYVRSRAWEGNGKYRTAHFNTTIQ

FKGNVRNLRIKLVEKTGLVWEPWRTVYDRSDLPLVRQRTISNWGTTLWRVAETVKNDHHHHHH

Experimental steps:

（1）PCR Amplification reaction system

F	1μl
R	1 μ l
Template	1 μ l (20-50ng)
10* pfu buffer	5μl
pfu	1 μ l (5U)
Add deionized water to	50μl

（2）PCR Amplification reaction conditions

Temperature	Time	Cycle
**95℃**	5min	
**95℃**	15s	
**55℃**	2Kb/min	**25**
**72℃**	1min	
**72℃**	10min	
**4℃**	∞	

Electrophoresis detection and recovery of amplified fragments should be performed according to the kit instructions.

S2.1.2 Vector and target gene digestion

Vector/Gene fragment	1-2 μg
10*cutsmart buffer	5 μl
EcoRI	1μl
NotI	1μl
Add deionized water to	50μl

37℃ 1-2h; Enzyme digestion products were detected by electrophoresis and recovered according to the kit instructions.

S2.1.3 Ligation of vector and target gene

Vector/Gene fragment	50ng
Target product	150ng
10*T4 DNA Ligase buffer	2μl
T4 DNA Ligase	1μl
Add deionized water to	20μl

16℃，0.5-1h

S2.1.4 Transformation

(1) Add the DNA fragment to be transformed to a tube containing TOP10 competent cells (50 μl of competent cells requires 25 ng of DNA). The volume should not exceed 5% of the competent cells. Gently vortex several times to mix the contents, then ice-bathe for 30 minutes.

(2) Place the centrifuge tube mixture into a water bath preheated to 42°C. Heat shock for 90 seconds without agitating the tube.

(3) Rapidly transfer the tube to an ice bath and cool the cells for 1–2 minutes.

(4) Add 200 μl of SOC liquid medium to each tube. Warm the medium to 37°C in a water bath, then transfer the tubes to a shaker set at 37°C and 220 rpm for 45 minutes to allow cell recovery and expression of the resistance marker gene encoded by the plasmid.

(5) Transfer an appropriate volume (up to 200 μl per 90 mm plate) of the transformed competent cells onto LB medium containing the corresponding antibiotic.

(6) Invert the plates and incubate at 37°C. Colony formation will be visible after 12–16 hours.

S2.1.5 Colony PCR Verification

Colonies grew on the plates. Several were randomly selected for colony PCR verification. Positive clones were sent to the sequencing platform for sequencing verification.

S2.1.6 Protein Expression Testing

E. coli expression system was employed for expression testing. The target gene was transformed into both BL21 and TE7 competent cells for protein expression testing.

Expression conditions:

- Induce protein expression when the strain reaches OD600 = 0.6
- Induction temperature: 18°C; 37°C
- Induction duration: 12 hours; 4 hours
- IPTG concentration: 1 mM

S2.1.7 Protein Purification test

Collect 2000 ml of culture supernatant, centrifuge at high speed, filter through a 0.22 μm membrane, and purify the target protein by binding it to Ni^{2+} resin.

Resin: Ni^{2+} resin.

Binding and equilibration buffer: PBS, pH=7.4. Wash and elution buffers:

Wash 1: PBS, pH=7.4;

Wash 2: PBS, pH=7.4, 30 mM imidazole;

Wash 3: PBS, pH=7.4, 50 mM imidazole;

Elution: PBS, pH=7.4, 300 mM imidazole.

S2.2 VLY Polyclonal Antibody Preparation

S2.2.1 Antigen processing

The primary antigen consists of a recombinant protein emulsified with an equal volume of complete Freund's adjuvant. The secondary, tertiary, and quaternary antigens consist of a recombinant protein emulsified with an equal volume of incomplete Freund's adjuvant.

S2.2.2 Animal immunization

Select healthy female New Zealand White rabbits.

Immunization Schedule:

First immunization: Immunizing antigen is Freund's complete adjuvant + 0.4mg recombinant protein;

Second immunization: Immunizing antigen is Freund's incomplete adjuvant + 0.2mg recombinant protein;

Third immunization: Immunizing antigen is Freund's incomplete adjuvant + 0.2mg recombinant protein; Fourth immunization: Immunization antigen: Freund's incomplete adjuvant + 0.2 mg recombinant protein; Fifth immunization: Immunization antigen: Freund's incomplete adjuvant + 0.2 mg recombinant protein; Final blood collection: When ELISA detection of antiserum titer meets requirements, collect whole blood via carotid artery.

S2.2.3 Indirect ELISA Detection

(1) Antigen Coating

Dilute antigen to 6 μg/ml in 0.05 M carbonate buffer (pH 9.6). Add 100 μl per well and incubate overnight at 4°C.

(2) Washing

Remove and wash three times with 0.05% Tween-20 (PBST), 3 minutes per wash.

(3) Blocking

Add 150 μl blocking solution (5% nonfat milk in PBST) to each well. Incubate at 37°C for 60 minutes.

(4) Washing

Remove wells and wash three times with 0.05% Tween-20 (PBST), 3 min/wash.

(5) Primary Antibody Incubation

Dilute the antiserum 1:1000, then perform serial dilutions. Incubate at 37°C for 1 hour.

(6) Washing

Remove and wash three times with 0.05% Tween-20 (PBST), 3 minutes per wash.

(7) Secondary Antibody Incubation

Add horseradish peroxidase-labeled goat anti-rabbit IgG (H+L), catalog #116154, from Jackson. Dilute 1:8000 and incubate at 37°C for 45 minutes.

(8) Wash the plate

Remove the plate and wash five times with 0.05% Tween-20 (PBST), 3 minutes per wash.

(9) Color development

Add 100 μl substrate solution (TMB) per well. Incubate for 5-10 minutes. Stop the reaction by adding 100 μl 2 mol/L sulfuric acid.

(10) OD measurement

Measure the optical density (OD) at 450 nm using an enzyme-linked immunosorbent assay reader (Kehua ST-360).

S2.2.4 Antibody affinity purification

(1) Place an appropriate amount of affinity purification beads into the purification column. Wash three times with 10 mM HCl (approximately 30 mL volume). Then wash with binding buffer to remove residual HCl. Add the purification antigen dissolved in PBS to the beads. After adding buffer, incubate overnight at 4°C. Subsequently, wash three times using the column-specific acid, water, and base in sequence. Finally, wash with PBS and set aside.

(2) Locate the frozen antiserum and corresponding beads according to the antibody purification schedule. Maintain detailed records.

(3) Thaw the serum and transfer the beads into the purification column. Measure the serum to the required volume and return any excess serum.

(4) Centrifuge the serum at 4-8°C at 3400 rpm for 20 minutes.

(5) Pipette off the lipid layer from the top of the centrifuged serum, reserving a small sample for ELISA testing.

(6) Incubate the centrifuged serum with the beads at room temperature for 2 hours (place in a 37°C incubator if room temperature is low) or overnight at 4°C.

(7) After incubation, decant the flow-through (FT). Wash beads three times with PBS, each wash volume 10 times the column volume.

(8) Pre-cool 5 ml pH 5.0 pre-wash buffer and wash the purification column.

(9) Elution: Elute with pre-chilled pH 2.5 elution buffer, collecting 1 ml per fraction. Add 50 µl neutralization buffer to each EP tube beforehand.

(10) Detect elution peaks using Coomassie staining. Add 10 µl eluate to 100 µl Coomassie medium. Identify target antibody based on elution peak and store at 4°C.

(11) Terminate collection when no color appears in Coomassie staining. Wash with 10 ml PBS.

(12) Re-incubate the eluate with beads and repeat steps 2.6–2.10.

(13) Concentrate the collected antibody using PEG.

S3 Experimental results

S3.1 VLYRecombinant protein expression and purification results

（1）Gene synthesis and vector construction were successful, enzyme digestion verification result (Figure S1）。

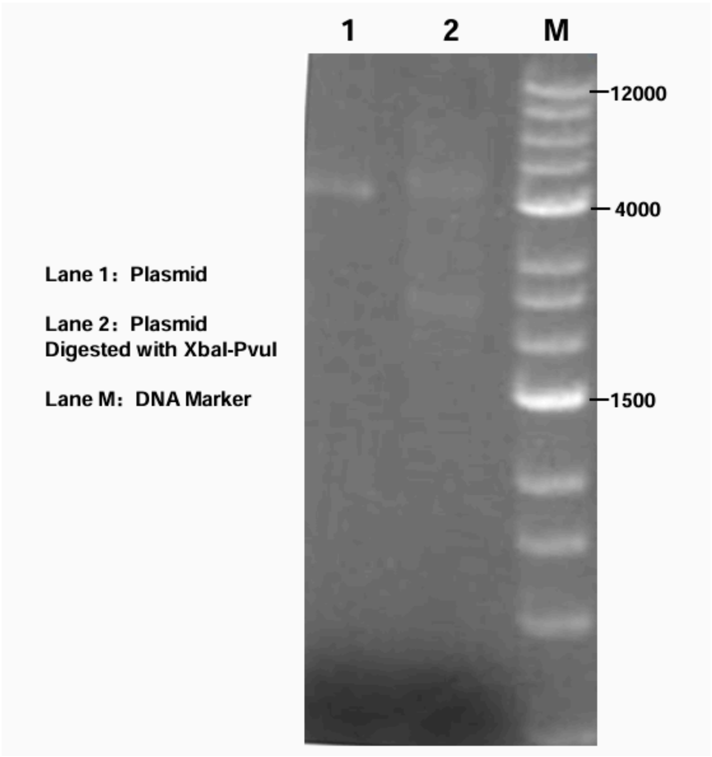

Figure S1. Enzyme digestion verification results of the constructed plasmid.

（2) proteinexpression test results

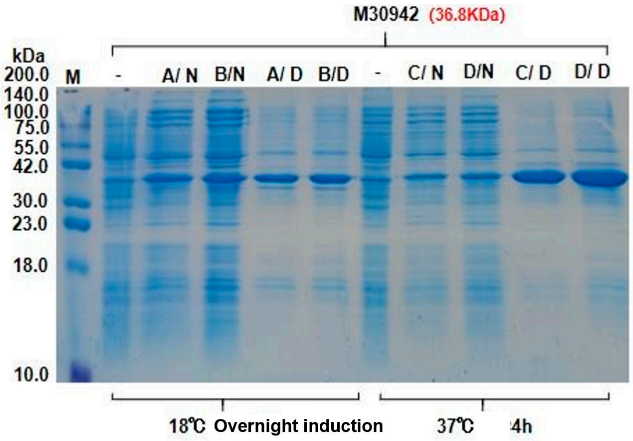

Figure S2. Target protein expression test (Coomassie brilliant blue staining).

M.proteinmarker -.Uninduced (negative control) A. T7E B. BL21 N. Supernatant after ultrasound

D. Sediment after ultrasound

Expression assays demonstrated that under 12-hour induction at 18°C, the target protein was expressed in the supernatant of both BL21 and T7E strains, with a molecular weight of approximately 36.8 kDa (Figure S2). The target protein was expressed in both competent cell strains and was detected in the supernatant.

（3）proteinpurification results

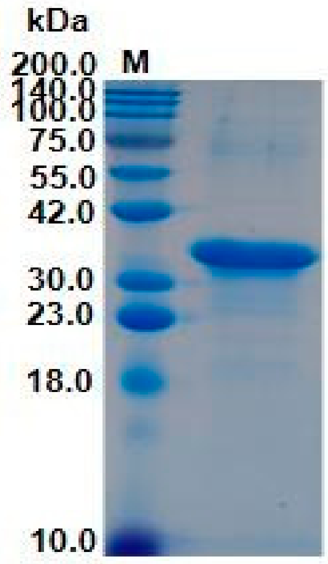

Figure S3. Target protein purification test (Coomassie brilliant blue staining).

Final expression level per liter: 1.5 mg/L, 18°C, 12 hours, purity >90%. Theoretical molecular weight calculated as 36.8 kDa, consistent with SDS-PAGE results. The protein is suitable for immunological applications.

S3.2 Polyclonal antibody preparation results

S3.2.1Antiserum titer detection

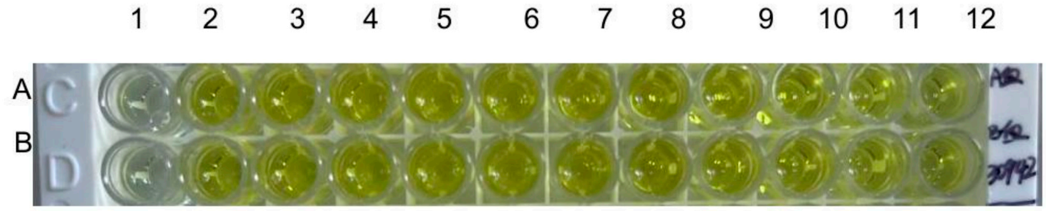

Figure S4. LISA test results.

Table S1. ELISA test data.

	Blank control	1K	2K	4K	8K	16K	32K	64K	128K	256K	512K	1024K
M30942-Aserum	0.211	1.21	1.169	1.23	1.153	1.141	1.053	1.1	0.92	0.81	0.76	0.421
M30942-Bserum	0.24	1.23	1.275	1.185	1.229	1.193	1.049	1.026	0.94	0.84	0.816	0.734

Final antibody titer after incubation: A ≥ 1024K, B ≥ 1024K.

As determined by ELISA assay, the antibody titer exceeded 512K, confirming successful antibody preparation.

S3.2.2 Western blot identification of polyclonal antibodies

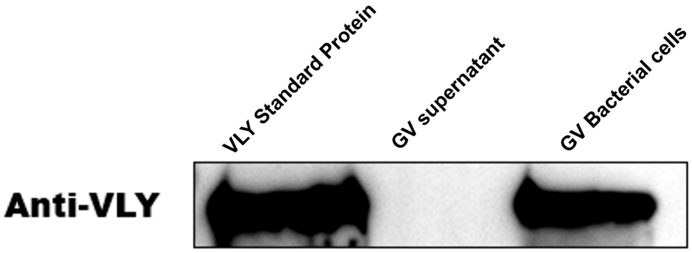

Figure S5. Polyclonal Antibody Western Blot Identification.

Western blot analysis revealed that the polyclonal antibody specifically recognized the VLY protein band with no significant non-specific bands observed (Figure S5). The antibody clearly identified the target band, demonstrating good specificity.
